# Poly[4,4′-imino­dipyridinium [di-μ_4_-isophthalato-κ^4^ *O*:*O*′:*O*′′:*O*′′-di-μ_3_-iso­phthal­ato-κ^3^ *O*:*O*′:*O*′′;κ^4^ *O*:*O*′:*O*′′,*O*′′′-trizinc(II)] dihydrate]

**DOI:** 10.1107/S1600536808038373

**Published:** 2008-11-22

**Authors:** Maxwell A. Braverman, Robert L. LaDuca

**Affiliations:** a Lyman Briggs College, Department of Chemistry, Michigan State University, East Lansing, MI 48825, USA

## Abstract

In the title compound, {(C_10_H_11_N_3_)[Zn_3_(C_8_H_4_O_4_)_4_]·2H_2_O}_*n*_, divalent Zn atoms are linked into trinuclear units featuring tetra­hedral, octa­hedral and distorted tetrahedral, octahedral and square-pyramidal coordination geometries. These trinuclear units are connected by isopthalate dianions into [Zn_3_(isophthalate)_4_]_*n*_^2*n*−^ anionic layers, which aggregate into the three-dimensional structure *via* hydrogen-bonding pathways mediated by doubly protonated 4,4′-imino­dipyridinium cations and water mol­ecules of crystallization. One solvent water mol­ecule was found to be disordered over two positions, each with a 50% site-occupancy factor.

## Related literature

For divalent metal phthalate/4,4′-imino­dipyridinium coordin­ation polymers, see: Braverman *et al.* (2007 ▶). For the preparation of 4,4′-dipyridylamine, see: Zapf *et al.* (1998 ▶). 
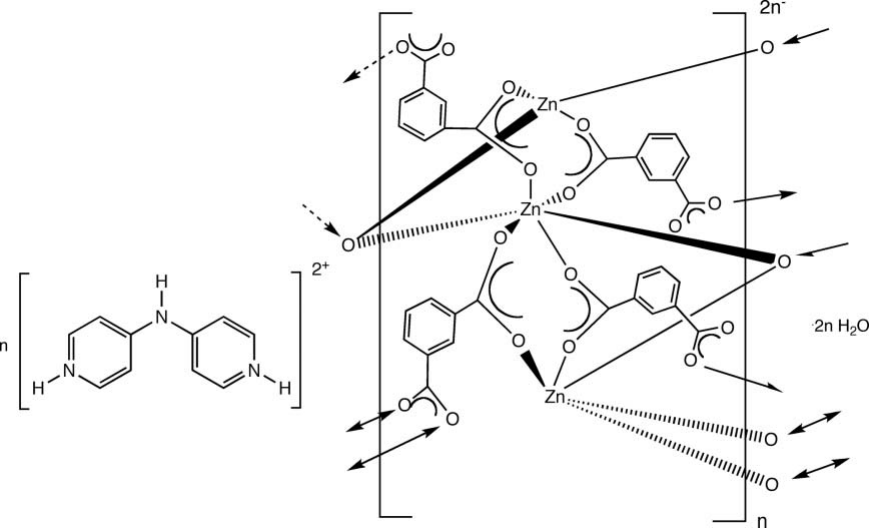


## Experimental

### 

#### Crystal data

(C_10_H_11_N_3_)[Zn_3_(C_8_H_4_O_4_)_4_]·2H_2_O
*M* *_r_* = 1061.81
Triclinic, 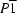

*a* = 9.5780 (13) Å
*b* = 10.2149 (14) Å
*c* = 21.246 (3) Å
α = 78.801 (2)°
β = 86.868 (2)°
γ = 87.773 (2)°
*V* = 2035.2 (5) Å^3^
*Z* = 2
Mo *K*α radiation
μ = 1.84 mm^−1^
*T* = 173 (2) K
0.54 × 0.20 × 0.12 mm

#### Data collection

Bruker SMART 1K diffractometer
Absorption correction: multi-scan (*SADABS*; Sheldrick, 1996 ▶) *T*_min_ = 0.617, *T*_max_ = 0.802
24469 measured reflections
9553 independent reflections
8108 reflections with *I* > 2σ(*I*)
*R*_int_ = 0.024

#### Refinement

*R*[*F* ^2^ > 2σ(*F* ^2^)] = 0.034
*wR*(*F* ^2^) = 0.081
*S* = 1.07
9553 reflections
619 parameters
6 restraints
H atoms treated by a mixture of independent and constrained refinement
Δρ_max_ = 0.92 e Å^−3^
Δρ_min_ = −0.54 e Å^−3^

### 

Data collection: *SMART* (Bruker, 2006 ▶); cell refinement: *SAINT* (Bruker, 2006 ▶); data reduction: *SAINT*; program(s) used to solve structure: *SHELXS97* (Sheldrick, 2008 ▶); program(s) used to refine structure: *SHELXL97* (Sheldrick, 2008 ▶); molecular graphics: *CrystalMaker* (Palmer, 2007 ▶); software used to prepare material for publication: *SHELXL97*.

## Supplementary Material

Crystal structure: contains datablocks I, global. DOI: 10.1107/S1600536808038373/tk2328sup1.cif

Structure factors: contains datablocks I. DOI: 10.1107/S1600536808038373/tk2328Isup2.hkl

Additional supplementary materials:  crystallographic information; 3D view; checkCIF report

## Figures and Tables

**Table 1:** Hydrogen-bond geometry (Å, °)

*D*—H⋯*A*	*D*—H	H⋯*A*	*D*⋯*A*	*D*—H⋯*A*
O1*W*—H1*A*⋯O8^i^	0.863 (18)	1.921 (19)	2.778 (3)	172 (4)
O1*W*—H1*B*⋯O4	0.863 (18)	1.874 (19)	2.733 (3)	174 (4)
N1—H1N⋯O2*WA*	0.901 (19)	2.03 (3)	2.808 (6)	144 (4)
N1—H1N⋯O2*WB*^ii^	0.901 (19)	1.96 (3)	2.757 (5)	147 (4)
N2—H2N⋯O1*W*^iii^	0.862 (17)	1.893 (18)	2.754 (3)	176 (3)
N3—H3N⋯O12^iv^	0.880 (17)	1.93 (2)	2.764 (3)	157 (3)

Symmetry codes: (i) 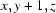; (ii) 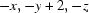; (iii) 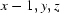; (iv) 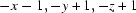.

**Fig. 1.**
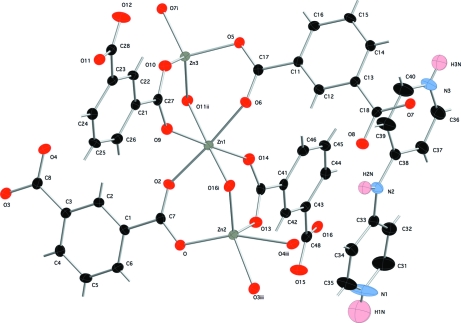
Coordination environment of (I), showing 50% probability ellipsoids and atom numbering scheme. Most hydrogen atom positions are shown as gray sticks. Unligated water molecules are not shown. Color codes: gray Zn, light blue N, red O, black C, pink H. Symmetry codes: (i) x - 1, y, z; (ii) x + 1, y, z; (iii) x, y - 1, z; (iv) x, y + 1, z

**Fig. 2.**
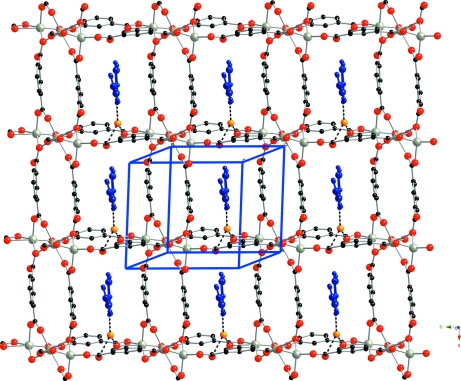
A single [Zn3(isophthalate)4]n2n- anionic layer in (I), showing [H2dpa]2+ cations within the incipient cavities in blue. Water molecules of crystallization are depicted in orange.

**Fig. 3.**
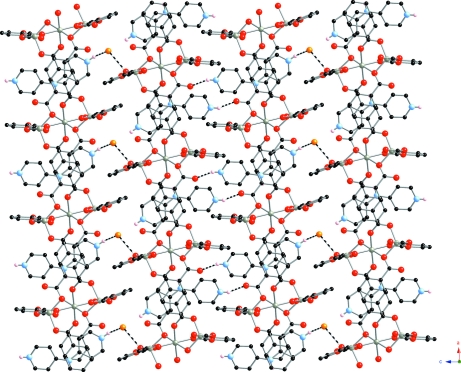
Packing diagram illustrating the AB layer stacking pattern, which forms the 3-D crystal structure of (I) through hydrogen bonding mediated by the protonated pyridyl groups and the amine groups of the [H2dpa]2+ cations. Hydrogen bonding is shown as dashed lines. The oxygen atoms of the water molecules of crystallization are shown in orange.

## supplementary crystallographic information

### Comment

Recently, we reported Co and Ni phthalate 1-D coordination polymers containing the hydrogen-bonding capable, dipodal tethering ligand 4,4'-dipyridylamine (dpa) (Braverman *et al.*, 2007). In an attempt to extend this chemistry into a zinc isophthalate coordination polymer system, colourless crystals of the title compound, (I), were obtained.

The asymmetric unit of (I) contains three divalent Zn atoms, four crystallographically distinct doubly deprotonated isophthalate dianions, one doubly protonated H_2_dpa^2+^ dication and two water molecules of crystallization, one of which is disordered equally over two positions, Fig. 1. The Zn atoms are linked into a trinuclear cluster by bridging carboxylate groups and O atoms from the isophthalate ions, in which Zn2, Zn1 and Zn3 adopt tetrahedral, octahedral and distorted square pyramidal coordination geometries, respectively.

Each trinuclear unit is linked to two others along the *a*-axis by two sets of two exotetradentate isophthalate dianions, which bridge two Zn atoms through a carboxylate bridge and two other Zn atoms through a single O atom connection. The trinuclear units also conjoin along the *b*-axis. Here, each trinuclear unit again connects to two others, *via* two sets of two crystallographically distinct exotridentate isophthalate dianions. One of these adopts a bis-bridging/chelating binding mode, while the other possesses a bis-bridging/monodentate binding mode. The resulting [Zn_3_(isophthalate)_4_]_n_^2n-^ anionic layers contain incipient voids occupied by H_2_dpa^2+^ dications and water molecules of crystallization, Fig. 2. These layers are arranged parallel to the *bc*-plane. Abutting [Zn_3_(isophthalate)_4_]_n_^2n-^ layers aggregate into the 3-D structure through hydrogen-bonding patterns between the protonated pyridyl-N atoms of the H_2_dpa^2+^ dications, carboxylate-O atoms and water molecules of crystallization, Table 1 and Fig. 3.

### Experimental

All chemicals were obtained commercially with the exception of 4,4'-dipyridylamine which was prepared according to a literature procedure (Zapf *et al.*, 1998). Zinc chloride dihydrate (64 mg, 0.37 mmol), isophthalic acid (62 mg, 0.37 mmol) and 4,4'-dipyridylamine (127 mg, 0.74 mmol) were placed into H_2_O (10 ml ) in a 23 ml Teflon-lined Parr acid digestion bomb. The bomb was heated at 393 K for 72 h and was then allowed to cool to room temperature. Colourless crystals of (I) were obtained along with a white powdery solid.

### Refinement

All H atoms bound to C atoms were placed in calculated positions, with C—H = 0.93 Å and refined in riding mode with *U*_iso_ = 1.2*U*_eq_(C). The H atoms bound to O atoms were found *via* a Fourier difference map, restrained at fixed positions or with O—H = 0.85 Å, and refined with *U*_iso_=1.2*U*_eq_(O). The H atoms bound to N atoms were found *via* a Fourier difference map, restrained with N—H = 0.91 Å (for pyridyl N atoms) or with N—H = 0.89 Å (for the amine N atom), and refined with *U*_iso_=1.2*U*_eq_(N). See Table 1 for O-H and N-H distances. One water molecule was found to be disorderd over two sites, each with equal weight; the H atoms could not be located.

### Crystal data

**Table:**

(C_10_H_11_N_3_)[Zn_3_(C_8_H_4_O_4_)_4_]·2H_2_O	*Z* = 2
*M**_r_* = 1061.81	*F*_000_ = 1072
Triclinic, *P*1	*D*_x_ = 1.729 Mg m^−^^3^
Hall symbol: -P 1	Mo *K*α radiation λ = 0.71073 Å
*a* = 9.5780 (13) Å	Cell parameters from 24469 reflections
*b* = 10.2149 (14) Å	θ = 2.0–28.3º
*c* = 21.246 (3) Å	µ = 1.84 mm^−^^1^
α = 78.801 (2)º	*T* = 173 (2) K
β = 86.868 (2)º	Block, colourless
γ = 87.773 (2)º	0.54 × 0.20 × 0.12 mm
*V* = 2035.2 (5) Å^3^	

### Data collection

**Table:**

Bruker SMART 1K diffractometer	9553 independent reflections
Radiation source: fine-focus sealed tube	8108 reflections with *I* > 2σ(*I*)
Monochromator: graphite	*R*_int_ = 0.024
*T* = 173(2) K	θ_max_ = 28.3º
ω scans	θ_min_ = 2.0º
Absorption correction: multi-scan(SADABS; Sheldrick, 1996)	*h* = −12→12
*T*_min_ = 0.617, *T*_max_ = 0.802	*k* = −13→13
24469 measured reflections	*l* = −28→27

### Refinement

**Table:**

Refinement on *F*^2^	Secondary atom site location: difference Fourier map
Least-squares matrix: full	Hydrogen site location: inferred from neighbouring sites
*R*[*F*^2^ > 2σ(*F*^2^)] = 0.034	H atoms treated by a mixture of independent and constrained refinement
*wR*(*F*^2^) = 0.081	*w* = 1/[σ^2^(*F*_o_^2^) + (0.0267*P*)^2^ + 2.909*P*] where *P* = (*F*_o_^2^ + 2*F*_c_^2^)/3
*S* = 1.07	(Δ/σ)_max_ < 0.001
9553 reflections	Δρ_max_ = 0.92 e Å^−^^3^
619 parameters	Δρ_min_ = −0.54 e Å^−^^3^
6 restraints	Extinction correction: none
Primary atom site location: structure-invariant direct methods	

### Special details

**Table:**

Geometry. All e.s.d.'s (except the e.s.d. in the dihedral angle between two l.s. planes) are estimated using the full covariance matrix. The cell e.s.d.'s are taken into account individually in the estimation of e.s.d.'s in distances, angles and torsion angles; correlations between e.s.d.'s in cell parameters are only used when they are defined by crystal symmetry. An approximate (isotropic) treatment of cell e.s.d.'s is used for estimating e.s.d.'s involving l.s. planes.
Refinement. Refinement of *F*^2^ against ALL reflections. The weighted *R*-factor *wR* and goodness of fit *S* are based on *F*^2^, conventional *R*-factors *R* are based on *F*, with *F* set to zero for negative *F*^2^. The threshold expression of *F*^2^ > σ(*F*^2^) is used only for calculating *R*-factors(gt) *etc*. and is not relevant to the choice of reflections for refinement. *R*-factors based on *F*^2^ are statistically about twice as large as those based on *F*, and *R*- factors based on ALL data will be even larger.

### Fractional atomic coordinates and isotropic or equivalent isotropic displacement parameters (Å^2^)

**Table:**

	*x*	*y*	*z*	*U*_iso_*/*U*_eq_	Occ. (<1)
Zn1	0.19902 (3)	0.26767 (3)	0.246490 (13)	0.01234 (6)	
Zn2	0.26205 (3)	0.12585 (3)	0.120459 (13)	0.01308 (7)	
Zn3	0.12868 (3)	0.40139 (3)	0.372471 (12)	0.01223 (7)	
O1	0.25397 (19)	0.31307 (16)	0.07148 (8)	0.0198 (4)	
O1W	0.3318 (2)	0.7891 (3)	0.26301 (11)	0.0473 (7)	
H1A	0.263 (3)	0.747 (4)	0.2846 (15)	0.057*	
H1B	0.304 (4)	0.816 (4)	0.2245 (10)	0.057*	
O2	0.24293 (18)	0.39914 (16)	0.16108 (8)	0.0163 (3)	
O2WB	0.0297 (4)	1.2705 (5)	−0.01008 (17)	0.0353 (10)	0.50
O3	0.3008 (2)	1.01970 (17)	0.04816 (8)	0.0221 (4)	
O2WA	0.0059 (6)	0.9448 (6)	0.0086 (2)	0.0554 (17)	0.50
O4	0.2498 (2)	0.89274 (18)	0.14176 (8)	0.0238 (4)	
O5	0.16648 (18)	0.22390 (16)	0.42778 (8)	0.0176 (4)	
O6	0.15301 (18)	0.14730 (16)	0.33667 (8)	0.0167 (3)	
O7	0.12225 (19)	−0.47631 (16)	0.43426 (8)	0.0177 (4)	
O8	0.1139 (2)	−0.33392 (17)	0.34139 (8)	0.0214 (4)	
O9	0.01511 (17)	0.36945 (17)	0.24508 (8)	0.0181 (4)	
O10	−0.06132 (17)	0.39961 (17)	0.34319 (8)	0.0169 (3)	
O11	−0.71777 (16)	0.41254 (16)	0.30577 (8)	0.0149 (3)	
O12	−0.58193 (19)	0.4146 (2)	0.38758 (8)	0.0268 (4)	
O13	0.45204 (17)	0.12092 (17)	0.15619 (8)	0.0181 (4)	
O14	0.38613 (17)	0.17370 (17)	0.25117 (8)	0.0179 (4)	
O15	0.9879 (2)	0.1151 (2)	0.11281 (9)	0.0270 (4)	
O16	1.11797 (17)	0.12825 (16)	0.19406 (8)	0.0156 (3)	
N1	−0.1435 (3)	0.8121 (4)	0.11834 (13)	0.0565 (10)	
H1N	−0.096 (4)	0.819 (4)	0.0802 (13)	0.068*	
N2	−0.3877 (2)	0.7558 (2)	0.28617 (10)	0.0184 (4)	
H2N	−0.4749 (19)	0.770 (3)	0.2783 (14)	0.022*	
N3	−0.3498 (2)	0.6593 (2)	0.48259 (11)	0.0261 (5)	
H3N	−0.346 (3)	0.635 (3)	0.5245 (9)	0.031*	
C1	0.2898 (3)	0.5447 (2)	0.06242 (11)	0.0159 (5)	
C2	0.2758 (3)	0.6576 (2)	0.09009 (11)	0.0158 (5)	
H2	0.2443	0.6497	0.1328	0.019*	
C3	0.3089 (3)	0.7831 (2)	0.05381 (11)	0.0162 (5)	
C4	0.3613 (3)	0.7939 (2)	−0.00916 (12)	0.0214 (5)	
H4	0.3863	0.8767	−0.0330	0.026*	
C5	0.3764 (3)	0.6809 (3)	−0.03659 (12)	0.0250 (6)	
H5	0.4120	0.6881	−0.0787	0.030*	
C6	0.3384 (3)	0.5574 (3)	−0.00129 (12)	0.0225 (5)	
H6	0.3455	0.4827	−0.0204	0.027*	
C7	0.2587 (2)	0.4086 (2)	0.10189 (11)	0.0146 (5)	
C8	0.2853 (3)	0.9050 (2)	0.08303 (12)	0.0163 (5)	
C11	0.1663 (2)	−0.0103 (2)	0.43341 (11)	0.0142 (4)	
C12	0.1514 (2)	−0.1155 (2)	0.40159 (11)	0.0148 (5)	
H12	0.1466	−0.0985	0.3571	0.018*	
C13	0.1436 (2)	−0.2461 (2)	0.43602 (11)	0.0139 (4)	
C14	0.1530 (3)	−0.2708 (2)	0.50250 (11)	0.0179 (5)	
H14	0.1470	−0.3577	0.5258	0.021*	
C15	0.1712 (3)	−0.1665 (2)	0.53413 (12)	0.0216 (5)	
H15	0.1796	−0.1837	0.5784	0.026*	
C16	0.1768 (3)	−0.0366 (2)	0.49976 (12)	0.0200 (5)	
H16	0.1877	0.0333	0.5212	0.024*	
C17	0.1636 (2)	0.1306 (2)	0.39605 (11)	0.0135 (4)	
C18	0.1254 (2)	−0.3577 (2)	0.40070 (11)	0.0138 (4)	
C21	−0.2241 (2)	0.4197 (2)	0.26180 (11)	0.0133 (4)	
C22	−0.3393 (2)	0.4109 (2)	0.30544 (11)	0.0138 (4)	
H22	−0.3264	0.3884	0.3493	0.017*	
C23	−0.4731 (2)	0.4356 (2)	0.28326 (11)	0.0136 (4)	
C24	−0.4919 (3)	0.4767 (2)	0.21774 (11)	0.0159 (5)	
H24	−0.5812	0.4978	0.2030	0.019*	
C25	−0.3769 (3)	0.4861 (2)	0.17443 (11)	0.0168 (5)	
H25	−0.3895	0.5137	0.1307	0.020*	
C26	−0.2433 (3)	0.4545 (2)	0.19603 (11)	0.0162 (5)	
H26	−0.1671	0.4566	0.1668	0.019*	
C27	−0.0791 (2)	0.3933 (2)	0.28499 (11)	0.0140 (4)	
C28	−0.5976 (2)	0.4193 (2)	0.32967 (11)	0.0150 (5)	
C31	−0.0791 (3)	0.7817 (4)	0.17389 (15)	0.0416 (8)	
H31	0.0181	0.7741	0.1731	0.050*	
C32	−0.1520 (3)	0.7618 (3)	0.23121 (13)	0.0246 (6)	
H32	−0.1054	0.7407	0.2693	0.030*	
C33	−0.2983 (3)	0.7733 (2)	0.23259 (12)	0.0184 (5)	
C34	−0.3620 (3)	0.8093 (3)	0.17313 (13)	0.0246 (6)	
H34	−0.4588	0.8202	0.1723	0.030*	
C35	−0.2832 (4)	0.8282 (4)	0.11717 (14)	0.0387 (8)	
H35	−0.3259	0.8522	0.0781	0.046*	
C36	−0.2372 (3)	0.6433 (3)	0.44465 (13)	0.0270 (6)	
H36	−0.1537	0.6113	0.4632	0.032*	
C37	−0.2419 (3)	0.6728 (3)	0.37923 (13)	0.0253 (6)	
H37	−0.1625	0.6596	0.3537	0.030*	
C38	−0.3657 (3)	0.7229 (2)	0.35070 (12)	0.0173 (5)	
C39	−0.4817 (3)	0.7387 (3)	0.39210 (13)	0.0327 (7)	
H39	−0.5664	0.7717	0.3752	0.039*	
C40	−0.4708 (3)	0.7060 (4)	0.45719 (14)	0.0376 (8)	
H40	−0.5486	0.7162	0.4842	0.045*	
C41	0.6209 (2)	0.1089 (2)	0.23487 (11)	0.0134 (4)	
C42	0.7377 (2)	0.1208 (2)	0.19237 (11)	0.0140 (4)	
H42	0.7264	0.1428	0.1483	0.017*	
C43	0.8713 (2)	0.0996 (2)	0.21573 (11)	0.0140 (4)	
C44	0.8875 (2)	0.0575 (2)	0.28136 (12)	0.0158 (5)	
H44	0.9766	0.0409	0.2970	0.019*	
C45	0.7707 (3)	0.0402 (2)	0.32346 (11)	0.0174 (5)	
H45	0.7816	0.0085	0.3671	0.021*	
C46	0.6379 (3)	0.0698 (2)	0.30081 (12)	0.0166 (5)	
H46	0.5604	0.0638	0.3294	0.020*	
C47	0.4750 (2)	0.1373 (2)	0.21204 (11)	0.0137 (4)	
C48	0.9977 (2)	0.1169 (2)	0.17032 (12)	0.0159 (5)	

### Atomic displacement parameters (Å^2^)

**Table:**

	*U*^11^	*U*^22^	*U*^33^	*U*^12^	*U*^13^	*U*^23^
Zn1	0.01115 (13)	0.01362 (13)	0.01198 (13)	0.00081 (10)	0.00085 (10)	−0.00242 (10)
Zn2	0.01370 (14)	0.01262 (13)	0.01368 (13)	−0.00055 (10)	−0.00062 (10)	−0.00445 (10)
Zn3	0.01145 (13)	0.01246 (13)	0.01351 (13)	−0.00087 (10)	−0.00011 (10)	−0.00432 (10)
O1	0.0310 (10)	0.0129 (8)	0.0157 (8)	0.0005 (7)	−0.0012 (7)	−0.0039 (7)
O1W	0.0225 (11)	0.0826 (19)	0.0267 (12)	−0.0079 (12)	−0.0048 (9)	0.0168 (12)
O2	0.0202 (9)	0.0156 (8)	0.0124 (8)	−0.0043 (7)	0.0009 (7)	−0.0010 (6)
O2WB	0.032 (2)	0.065 (3)	0.0106 (17)	−0.002 (2)	0.0014 (16)	−0.0108 (18)
O3	0.0345 (11)	0.0132 (8)	0.0194 (9)	−0.0016 (7)	0.0004 (8)	−0.0051 (7)
O2WA	0.045 (3)	0.099 (5)	0.026 (3)	−0.019 (4)	0.005 (2)	−0.020 (3)
O4	0.0344 (11)	0.0198 (9)	0.0183 (9)	0.0018 (8)	0.0026 (8)	−0.0081 (7)
O5	0.0249 (9)	0.0117 (8)	0.0170 (8)	−0.0014 (7)	−0.0015 (7)	−0.0042 (6)
O6	0.0215 (9)	0.0150 (8)	0.0134 (8)	−0.0041 (7)	−0.0018 (7)	−0.0013 (6)
O7	0.0257 (9)	0.0121 (8)	0.0158 (8)	−0.0008 (7)	−0.0003 (7)	−0.0039 (6)
O8	0.0312 (10)	0.0185 (9)	0.0152 (8)	−0.0039 (8)	−0.0011 (7)	−0.0043 (7)
O9	0.0120 (8)	0.0227 (9)	0.0184 (9)	0.0038 (7)	0.0024 (7)	−0.0025 (7)
O10	0.0113 (8)	0.0234 (9)	0.0170 (8)	−0.0015 (7)	−0.0014 (6)	−0.0060 (7)
O11	0.0106 (8)	0.0171 (8)	0.0174 (8)	−0.0016 (6)	0.0007 (6)	−0.0043 (7)
O12	0.0174 (9)	0.0480 (12)	0.0158 (9)	−0.0067 (8)	0.0021 (7)	−0.0078 (8)
O13	0.0141 (8)	0.0237 (9)	0.0174 (8)	−0.0005 (7)	−0.0016 (7)	−0.0061 (7)
O14	0.0129 (8)	0.0211 (9)	0.0205 (9)	0.0025 (7)	−0.0023 (7)	−0.0064 (7)
O15	0.0216 (10)	0.0446 (12)	0.0166 (9)	−0.0027 (8)	0.0013 (7)	−0.0103 (8)
O16	0.0112 (8)	0.0150 (8)	0.0214 (9)	−0.0012 (6)	0.0010 (7)	−0.0062 (7)
N1	0.0381 (17)	0.114 (3)	0.0187 (13)	−0.0153 (18)	0.0111 (12)	−0.0176 (16)
N2	0.0169 (10)	0.0220 (11)	0.0156 (10)	−0.0020 (8)	−0.0008 (8)	−0.0015 (8)
N3	0.0263 (12)	0.0364 (13)	0.0147 (11)	−0.0025 (10)	−0.0025 (9)	−0.0020 (10)
C1	0.0201 (12)	0.0134 (11)	0.0130 (11)	−0.0002 (9)	0.0001 (9)	0.0000 (9)
C2	0.0182 (12)	0.0160 (11)	0.0130 (11)	0.0010 (9)	0.0008 (9)	−0.0030 (9)
C3	0.0194 (12)	0.0141 (11)	0.0157 (11)	0.0004 (9)	−0.0014 (9)	−0.0044 (9)
C4	0.0323 (15)	0.0115 (11)	0.0185 (12)	0.0001 (10)	0.0038 (11)	0.0005 (9)
C5	0.0417 (17)	0.0186 (13)	0.0131 (12)	0.0007 (11)	0.0081 (11)	−0.0020 (10)
C6	0.0365 (15)	0.0153 (12)	0.0158 (12)	0.0008 (11)	0.0035 (11)	−0.0051 (9)
C7	0.0138 (11)	0.0135 (11)	0.0158 (11)	0.0007 (9)	−0.0016 (9)	−0.0007 (9)
C8	0.0165 (12)	0.0143 (11)	0.0193 (12)	0.0007 (9)	−0.0044 (9)	−0.0056 (9)
C11	0.0153 (11)	0.0122 (11)	0.0150 (11)	−0.0001 (9)	−0.0004 (9)	−0.0024 (9)
C12	0.0146 (11)	0.0157 (11)	0.0139 (11)	−0.0001 (9)	−0.0007 (9)	−0.0021 (9)
C13	0.0135 (11)	0.0132 (11)	0.0155 (11)	−0.0006 (8)	0.0000 (9)	−0.0039 (9)
C14	0.0252 (13)	0.0124 (11)	0.0152 (11)	−0.0012 (9)	0.0012 (10)	−0.0011 (9)
C15	0.0362 (15)	0.0177 (12)	0.0106 (11)	−0.0022 (11)	−0.0013 (10)	−0.0016 (9)
C16	0.0314 (14)	0.0150 (11)	0.0150 (11)	−0.0022 (10)	−0.0011 (10)	−0.0061 (9)
C17	0.0101 (11)	0.0136 (11)	0.0163 (11)	−0.0013 (8)	−0.0001 (9)	−0.0016 (9)
C18	0.0118 (11)	0.0128 (11)	0.0170 (11)	−0.0020 (8)	0.0025 (9)	−0.0041 (9)
C21	0.0123 (11)	0.0111 (10)	0.0166 (11)	−0.0007 (8)	−0.0004 (9)	−0.0032 (9)
C22	0.0151 (11)	0.0152 (11)	0.0116 (10)	−0.0018 (9)	−0.0012 (9)	−0.0030 (9)
C23	0.0133 (11)	0.0121 (10)	0.0158 (11)	−0.0022 (8)	0.0015 (9)	−0.0040 (9)
C24	0.0132 (11)	0.0165 (11)	0.0181 (12)	−0.0010 (9)	−0.0018 (9)	−0.0032 (9)
C25	0.0191 (12)	0.0203 (12)	0.0110 (11)	−0.0017 (9)	−0.0024 (9)	−0.0020 (9)
C26	0.0148 (11)	0.0173 (11)	0.0168 (11)	−0.0018 (9)	0.0035 (9)	−0.0047 (9)
C27	0.0138 (11)	0.0093 (10)	0.0188 (11)	−0.0023 (8)	0.0003 (9)	−0.0024 (9)
C28	0.0138 (11)	0.0143 (11)	0.0169 (11)	−0.0011 (9)	0.0012 (9)	−0.0036 (9)
C31	0.0277 (16)	0.069 (2)	0.0312 (17)	−0.0091 (16)	0.0051 (13)	−0.0172 (16)
C32	0.0250 (14)	0.0270 (14)	0.0231 (13)	−0.0051 (11)	−0.0018 (11)	−0.0071 (11)
C33	0.0265 (13)	0.0106 (11)	0.0186 (12)	−0.0045 (9)	0.0011 (10)	−0.0035 (9)
C34	0.0292 (15)	0.0229 (13)	0.0214 (13)	−0.0001 (11)	−0.0023 (11)	−0.0032 (10)
C35	0.0403 (19)	0.058 (2)	0.0184 (14)	−0.0066 (16)	−0.0017 (13)	−0.0067 (14)
C36	0.0232 (14)	0.0327 (15)	0.0224 (13)	0.0026 (11)	−0.0030 (11)	0.0010 (11)
C37	0.0216 (13)	0.0315 (15)	0.0202 (13)	0.0056 (11)	0.0014 (11)	−0.0001 (11)
C38	0.0199 (12)	0.0151 (11)	0.0166 (12)	−0.0025 (9)	−0.0016 (10)	−0.0022 (9)
C39	0.0151 (13)	0.061 (2)	0.0211 (14)	0.0032 (13)	−0.0022 (11)	−0.0054 (13)
C40	0.0201 (15)	0.069 (2)	0.0217 (14)	0.0003 (14)	0.0023 (11)	−0.0058 (14)
C41	0.0126 (11)	0.0103 (10)	0.0180 (11)	−0.0003 (8)	−0.0035 (9)	−0.0033 (9)
C42	0.0162 (11)	0.0131 (11)	0.0135 (11)	−0.0024 (9)	−0.0013 (9)	−0.0039 (9)
C43	0.0136 (11)	0.0118 (10)	0.0175 (11)	−0.0010 (8)	0.0002 (9)	−0.0055 (9)
C44	0.0141 (11)	0.0147 (11)	0.0200 (12)	−0.0013 (9)	−0.0067 (9)	−0.0046 (9)
C45	0.0214 (13)	0.0175 (12)	0.0135 (11)	−0.0026 (10)	−0.0030 (9)	−0.0026 (9)
C46	0.0164 (12)	0.0164 (11)	0.0172 (12)	−0.0019 (9)	0.0020 (9)	−0.0043 (9)
C47	0.0122 (11)	0.0098 (10)	0.0183 (11)	−0.0022 (8)	0.0009 (9)	−0.0008 (9)
C48	0.0142 (11)	0.0143 (11)	0.0194 (12)	−0.0011 (9)	0.0016 (9)	−0.0046 (9)

### Geometric parameters (Å, °)

**Table:**

Zn1—O14	1.9980 (17)	C6—H6	0.9300
Zn1—O9	2.0089 (17)	C11—C12	1.391 (3)
Zn1—O2	2.0705 (16)	C11—C16	1.391 (3)
Zn1—O6	2.1005 (16)	C11—C17	1.503 (3)
Zn1—O16^i^	2.1607 (16)	C12—C13	1.394 (3)
Zn1—O11^ii^	2.3100 (16)	C12—H12	0.9300
Zn2—O1	1.9927 (17)	C13—C14	1.393 (3)
Zn2—O13	2.0048 (17)	C13—C18	1.502 (3)
Zn2—O16^i^	2.0314 (17)	C14—C15	1.387 (3)
Zn2—O3^iii^	2.0559 (17)	C14—H14	0.9300
Zn2—O4^iii^	2.3422 (18)	C15—C16	1.386 (3)
Zn3—O10	1.9569 (16)	C15—H15	0.9300
Zn3—O11^ii^	1.9766 (16)	C16—H16	0.9300
Zn3—O7^iv^	1.9773 (16)	C21—C26	1.394 (3)
Zn3—O5	1.9917 (17)	C21—C22	1.396 (3)
O1—C7	1.274 (3)	C21—C27	1.496 (3)
O1W—H1A	0.863 (18)	C22—C23	1.386 (3)
O1W—H1B	0.863 (18)	C22—H22	0.9300
O2—C7	1.244 (3)	C23—C24	1.393 (3)
O3—C8	1.268 (3)	C23—C28	1.499 (3)
O4—C8	1.259 (3)	C24—C25	1.390 (3)
O5—C17	1.272 (3)	C24—H24	0.9300
O6—C17	1.249 (3)	C25—C26	1.388 (3)
O7—C18	1.281 (3)	C25—H25	0.9300
O8—C18	1.246 (3)	C26—H26	0.9300
O9—C27	1.253 (3)	C31—C32	1.354 (4)
O10—C27	1.272 (3)	C31—H31	0.9300
O11—C28	1.293 (3)	C32—C33	1.401 (4)
O12—C28	1.239 (3)	C32—H32	0.9300
O13—C47	1.262 (3)	C33—C34	1.411 (4)
O14—C47	1.254 (3)	C34—C35	1.358 (4)
O15—C48	1.234 (3)	C34—H34	0.9300
O16—C48	1.301 (3)	C35—H35	0.9300
N1—C31	1.339 (4)	C36—C37	1.367 (4)
N1—C35	1.343 (4)	C36—H36	0.9300
N1—H1N	0.901 (19)	C37—C38	1.396 (4)
N2—C38	1.373 (3)	C37—H37	0.9300
N2—C33	1.374 (3)	C38—C39	1.403 (4)
N2—H2N	0.862 (17)	C39—C40	1.367 (4)
N3—C36	1.335 (4)	C39—H39	0.9300
N3—C40	1.337 (4)	C40—H40	0.9300
N3—H3N	0.880 (17)	C41—C42	1.392 (3)
C1—C6	1.390 (3)	C41—C46	1.397 (3)
C1—C2	1.392 (3)	C41—C47	1.503 (3)
C1—C7	1.509 (3)	C42—C43	1.392 (3)
C2—C3	1.400 (3)	C42—H42	0.9300
C2—H2	0.9300	C43—C44	1.393 (3)
C3—C4	1.388 (3)	C43—C48	1.500 (3)
C3—C8	1.500 (3)	C44—C45	1.388 (3)
C4—C5	1.390 (4)	C44—H44	0.9300
C4—H4	0.9300	C45—C46	1.386 (3)
C5—C6	1.387 (3)	C45—H45	0.9300
C5—H5	0.9300	C46—H46	0.9300
			
O14—Zn1—O9	176.55 (7)	C15—C14—C13	120.3 (2)
O14—Zn1—O2	96.23 (7)	C15—C14—H14	119.9
O9—Zn1—O2	84.01 (7)	C13—C14—H14	119.9
O14—Zn1—O6	85.68 (7)	C16—C15—C14	120.0 (2)
O9—Zn1—O6	93.84 (7)	C16—C15—H15	120.0
O2—Zn1—O6	175.51 (6)	C14—C15—H15	120.0
O14—Zn1—O16^i^	91.83 (7)	C15—C16—C11	120.3 (2)
O9—Zn1—O16^i^	91.61 (7)	C15—C16—H16	119.8
O2—Zn1—O16^i^	90.24 (6)	C11—C16—H16	119.8
O6—Zn1—O16^i^	93.76 (6)	O6—C17—O5	125.0 (2)
O14—Zn1—O11^ii^	88.41 (6)	O6—C17—C11	117.7 (2)
O9—Zn1—O11^ii^	88.14 (7)	O5—C17—C11	117.3 (2)
O2—Zn1—O11^ii^	91.68 (6)	O8—C18—O7	122.5 (2)
O6—Zn1—O11^ii^	84.30 (6)	O8—C18—C13	120.5 (2)
O16^i^—Zn1—O11^ii^	178.03 (6)	O7—C18—C13	117.0 (2)
O1—Zn2—O13	100.18 (7)	C26—C21—C22	120.1 (2)
O1—Zn2—O16^i^	103.26 (7)	C26—C21—C27	119.4 (2)
O13—Zn2—O16^i^	107.64 (7)	C22—C21—C27	120.6 (2)
O1—Zn2—O3^iii^	101.82 (7)	C23—C22—C21	119.9 (2)
O13—Zn2—O3^iii^	101.12 (7)	C23—C22—H22	120.0
O16^i^—Zn2—O3^iii^	137.35 (7)	C21—C22—H22	120.0
O1—Zn2—O4^iii^	159.21 (7)	C22—C23—C24	120.0 (2)
O13—Zn2—O4^iii^	92.86 (7)	C22—C23—C28	120.1 (2)
O16^i^—Zn2—O4^iii^	87.90 (6)	C24—C23—C28	119.9 (2)
O3^iii^—Zn2—O4^iii^	59.44 (6)	C25—C24—C23	119.9 (2)
O10—Zn3—O11^ii^	116.47 (7)	C25—C24—H24	120.1
O10—Zn3—O7^iv^	106.51 (7)	C23—C24—H24	120.1
O11^ii^—Zn3—O7^iv^	119.79 (7)	C26—C25—C24	120.4 (2)
O10—Zn3—O5	107.06 (7)	C26—C25—H25	119.8
O11^ii^—Zn3—O5	102.47 (7)	C24—C25—H25	119.8
O7^iv^—Zn3—O5	102.88 (7)	C25—C26—C21	119.6 (2)
C7—O1—Zn2	118.93 (15)	C25—C26—H26	120.2
H1A—O1W—H1B	106 (3)	C21—C26—H26	120.2
C7—O2—Zn1	143.82 (16)	O9—C27—O10	125.5 (2)
C8—O3—Zn2^iv^	96.33 (14)	O9—C27—C21	117.3 (2)
C8—O4—Zn2^iv^	83.55 (14)	O10—C27—C21	117.2 (2)
C17—O5—Zn3	111.28 (15)	O12—C28—O11	123.6 (2)
C17—O6—Zn1	146.85 (15)	O12—C28—C23	119.8 (2)
C18—O7—Zn3^iii^	106.38 (14)	O11—C28—C23	116.6 (2)
C27—O9—Zn1	137.38 (16)	N1—C31—C32	121.6 (3)
C27—O10—Zn3	119.50 (15)	N1—C31—H31	119.2
C28—O11—Zn3^i^	111.44 (14)	C32—C31—H31	119.2
C28—O11—Zn1^i^	131.84 (15)	C31—C32—C33	119.4 (3)
Zn3^i^—O11—Zn1^i^	98.76 (7)	C31—C32—H32	120.3
C47—O13—Zn2	124.81 (15)	C33—C32—H32	120.3
C47—O14—Zn1	136.38 (16)	N2—C33—C32	126.9 (2)
C48—O16—Zn2^ii^	105.49 (14)	N2—C33—C34	115.8 (2)
C48—O16—Zn1^ii^	133.79 (15)	C32—C33—C34	117.3 (2)
Zn2^ii^—O16—Zn1^ii^	103.84 (7)	C35—C34—C33	120.6 (3)
C31—N1—C35	121.3 (3)	C35—C34—H34	119.7
C31—N1—H1N	122 (3)	C33—C34—H34	119.7
C35—N1—H1N	117 (3)	N1—C35—C34	119.8 (3)
C38—N2—C33	132.6 (2)	N1—C35—H35	120.1
C38—N2—H2N	113 (2)	C34—C35—H35	120.1
C33—N2—H2N	115 (2)	N3—C36—C37	121.5 (3)
C36—N3—C40	120.4 (2)	N3—C36—H36	119.3
C36—N3—H3N	120 (2)	C37—C36—H36	119.3
C40—N3—H3N	119 (2)	C36—C37—C38	120.0 (3)
C6—C1—C2	119.4 (2)	C36—C37—H37	120.0
C6—C1—C7	120.1 (2)	C38—C37—H37	120.0
C2—C1—C7	120.4 (2)	N2—C38—C37	127.0 (2)
C1—C2—C3	120.2 (2)	N2—C38—C39	116.1 (2)
C1—C2—H2	119.9	C37—C38—C39	116.9 (2)
C3—C2—H2	119.9	C40—C39—C38	120.3 (3)
C4—C3—C2	119.8 (2)	C40—C39—H39	119.8
C4—C3—C8	120.6 (2)	C38—C39—H39	119.8
C2—C3—C8	119.7 (2)	N3—C40—C39	120.9 (3)
C3—C4—C5	119.9 (2)	N3—C40—H40	119.6
C3—C4—H4	120.0	C39—C40—H40	119.6
C5—C4—H4	120.0	C42—C41—C46	119.8 (2)
C6—C5—C4	120.1 (2)	C42—C41—C47	121.9 (2)
C6—C5—H5	119.9	C46—C41—C47	118.2 (2)
C4—C5—H5	119.9	C43—C42—C41	120.1 (2)
C5—C6—C1	120.4 (2)	C43—C42—H42	120.0
C5—C6—H6	119.8	C41—C42—H42	120.0
C1—C6—H6	119.8	C42—C43—C44	119.8 (2)
O2—C7—O1	125.9 (2)	C42—C43—C48	120.3 (2)
O2—C7—C1	117.3 (2)	C44—C43—C48	119.9 (2)
O1—C7—C1	116.8 (2)	C45—C44—C43	120.0 (2)
O4—C8—O3	120.6 (2)	C45—C44—H44	120.0
O4—C8—C3	119.8 (2)	C43—C44—H44	120.0
O3—C8—C3	119.6 (2)	C46—C45—C44	120.3 (2)
O4—C8—Zn2^iv^	66.84 (13)	C46—C45—H45	119.9
O3—C8—Zn2^iv^	53.82 (12)	C44—C45—H45	119.9
C3—C8—Zn2^iv^	173.03 (18)	C45—C46—C41	119.8 (2)
C12—C11—C16	119.6 (2)	C45—C46—H46	120.1
C12—C11—C17	119.5 (2)	C41—C46—H46	120.1
C16—C11—C17	120.9 (2)	O14—C47—O13	125.8 (2)
C11—C12—C13	120.3 (2)	O14—C47—C41	116.3 (2)
C11—C12—H12	119.8	O13—C47—C41	117.9 (2)
C13—C12—H12	119.8	O15—C48—O16	121.4 (2)
C14—C13—C12	119.5 (2)	O15—C48—C43	120.8 (2)
C14—C13—C18	121.2 (2)	O16—C48—C43	117.8 (2)
C12—C13—C18	119.4 (2)		
			
O13—Zn2—O1—C7	−62.52 (19)	Zn3^iii^—O7—C18—C13	−170.95 (16)
O16^i^—Zn2—O1—C7	48.50 (19)	C14—C13—C18—O8	178.4 (2)
O3^iii^—Zn2—O1—C7	−166.28 (18)	C12—C13—C18—O8	−1.7 (3)
O4^iii^—Zn2—O1—C7	169.47 (18)	C14—C13—C18—O7	−1.6 (3)
C8^iii^—Zn2—O1—C7	−171.67 (16)	C12—C13—C18—O7	178.3 (2)
O14—Zn1—O2—C7	74.6 (3)	C26—C21—C22—C23	0.6 (3)
O9—Zn1—O2—C7	−108.8 (3)	C27—C21—C22—C23	−180.0 (2)
O16^i^—Zn1—O2—C7	−17.2 (3)	C21—C22—C23—C24	−3.7 (3)
O11^ii^—Zn1—O2—C7	163.2 (3)	C21—C22—C23—C28	176.8 (2)
O10—Zn3—O5—C17	63.03 (17)	C22—C23—C24—C25	3.4 (3)
O11^ii^—Zn3—O5—C17	−60.01 (16)	C28—C23—C24—C25	−177.2 (2)
O7^iv^—Zn3—O5—C17	175.06 (15)	C23—C24—C25—C26	0.1 (4)
O14—Zn1—O6—C17	82.3 (3)	C24—C25—C26—C21	−3.2 (4)
O9—Zn1—O6—C17	−94.3 (3)	C22—C21—C26—C25	2.9 (3)
O16^i^—Zn1—O6—C17	173.8 (3)	C27—C21—C26—C25	−176.6 (2)
O11^ii^—Zn1—O6—C17	−6.6 (3)	Zn1—O9—C27—O10	29.2 (4)
O2—Zn1—O9—C27	−155.4 (2)	Zn1—O9—C27—C21	−152.36 (18)
O6—Zn1—O9—C27	20.6 (2)	Zn3—O10—C27—O9	10.4 (3)
O16^i^—Zn1—O9—C27	114.5 (2)	Zn3—O10—C27—C21	−168.02 (15)
O11^ii^—Zn1—O9—C27	−63.5 (2)	C26—C21—C27—O9	−18.2 (3)
O11^ii^—Zn3—O10—C27	6.36 (19)	C22—C21—C27—O9	162.4 (2)
O7^iv^—Zn3—O10—C27	142.94 (16)	C26—C21—C27—O10	160.4 (2)
O5—Zn3—O10—C27	−107.54 (17)	C22—C21—C27—O10	−19.0 (3)
O1—Zn2—O13—C47	97.71 (19)	Zn3^i^—O11—C28—O12	2.3 (3)
O16^i^—Zn2—O13—C47	−9.8 (2)	Zn1^i^—O11—C28—O12	−122.5 (2)
O3^iii^—Zn2—O13—C47	−157.96 (18)	Zn3^i^—O11—C28—C23	−176.84 (15)
O4^iii^—Zn2—O13—C47	−98.55 (18)	Zn1^i^—O11—C28—C23	58.3 (3)
C8^iii^—Zn2—O13—C47	−127.97 (19)	C22—C23—C28—O12	16.8 (3)
O2—Zn1—O14—C47	−37.1 (2)	C24—C23—C28—O12	−162.7 (2)
O6—Zn1—O14—C47	147.0 (2)	C22—C23—C28—O11	−164.0 (2)
O16^i^—Zn1—O14—C47	53.3 (2)	C24—C23—C28—O11	16.5 (3)
O11^ii^—Zn1—O14—C47	−128.6 (2)	C35—N1—C31—C32	2.1 (6)
C6—C1—C2—C3	0.7 (4)	N1—C31—C32—C33	−0.1 (5)
C7—C1—C2—C3	178.0 (2)	C38—N2—C33—C32	−0.5 (4)
C1—C2—C3—C4	−2.6 (4)	C38—N2—C33—C34	−179.2 (2)
C1—C2—C3—C8	176.4 (2)	C31—C32—C33—N2	179.6 (3)
C2—C3—C4—C5	2.1 (4)	C31—C32—C33—C34	−1.8 (4)
C8—C3—C4—C5	−176.9 (2)	N2—C33—C34—C35	−179.5 (3)
C3—C4—C5—C6	0.4 (4)	C32—C33—C34—C35	1.7 (4)
C4—C5—C6—C1	−2.3 (4)	C31—N1—C35—C34	−2.1 (6)
C2—C1—C6—C5	1.8 (4)	C33—C34—C35—N1	0.2 (5)
C7—C1—C6—C5	−175.6 (2)	C40—N3—C36—C37	−0.4 (5)
Zn1—O2—C7—O1	−2.7 (4)	N3—C36—C37—C38	1.0 (4)
Zn1—O2—C7—C1	178.93 (18)	C33—N2—C38—C37	−13.3 (4)
Zn2—O1—C7—O2	−13.8 (3)	C33—N2—C38—C39	168.3 (3)
Zn2—O1—C7—C1	164.62 (16)	C36—C37—C38—N2	−179.2 (3)
C6—C1—C7—O2	165.9 (2)	C36—C37—C38—C39	−0.8 (4)
C2—C1—C7—O2	−11.4 (3)	N2—C38—C39—C40	178.7 (3)
C6—C1—C7—O1	−12.6 (3)	C37—C38—C39—C40	0.1 (4)
C2—C1—C7—O1	170.1 (2)	C36—N3—C40—C39	−0.3 (5)
Zn2^iv^—O4—C8—O3	−2.6 (2)	C38—C39—C40—N3	0.5 (5)
Zn2^iv^—O4—C8—C3	177.7 (2)	C46—C41—C42—C43	−2.9 (3)
Zn2^iv^—O3—C8—O4	3.0 (3)	C47—C41—C42—C43	177.0 (2)
Zn2^iv^—O3—C8—C3	−177.37 (19)	C41—C42—C43—C44	4.5 (3)
C4—C3—C8—O4	−173.6 (2)	C41—C42—C43—C48	−177.9 (2)
C2—C3—C8—O4	7.3 (4)	C42—C43—C44—C45	−1.8 (3)
C4—C3—C8—O3	6.7 (4)	C48—C43—C44—C45	−179.4 (2)
C2—C3—C8—O3	−172.4 (2)	C43—C44—C45—C46	−2.6 (3)
C16—C11—C12—C13	1.5 (4)	C44—C45—C46—C41	4.2 (4)
C17—C11—C12—C13	−175.4 (2)	C42—C41—C46—C45	−1.5 (3)
C11—C12—C13—C14	−0.9 (3)	C47—C41—C46—C45	178.7 (2)
C11—C12—C13—C18	179.1 (2)	Zn1—O14—C47—O13	−20.3 (4)
C12—C13—C14—C15	−0.6 (4)	Zn1—O14—C47—C41	160.78 (16)
C18—C13—C14—C15	179.4 (2)	Zn2—O13—C47—O14	−8.5 (3)
C13—C14—C15—C16	1.5 (4)	Zn2—O13—C47—C41	170.41 (15)
C14—C15—C16—C11	−0.9 (4)	C42—C41—C47—O14	−153.6 (2)
C12—C11—C16—C15	−0.6 (4)	C46—C41—C47—O14	26.3 (3)
C17—C11—C16—C15	176.3 (2)	C42—C41—C47—O13	27.4 (3)
Zn1—O6—C17—O5	32.2 (4)	C46—C41—C47—O13	−152.7 (2)
Zn1—O6—C17—C11	−150.8 (2)	Zn2^ii^—O16—C48—O15	−4.2 (3)
Zn3—O5—C17—O6	7.0 (3)	Zn1^ii^—O16—C48—O15	123.4 (2)
Zn3—O5—C17—C11	−170.05 (15)	Zn2^ii^—O16—C48—C43	173.05 (16)
C12—C11—C17—O6	−2.6 (3)	Zn1^ii^—O16—C48—C43	−59.4 (3)
C16—C11—C17—O6	−179.5 (2)	C42—C43—C48—O15	−19.1 (3)
C12—C11—C17—O5	174.6 (2)	C44—C43—C48—O15	158.5 (2)
C16—C11—C17—O5	−2.3 (3)	C42—C43—C48—O16	163.6 (2)
Zn3^iii^—O7—C18—O8	9.1 (3)	C44—C43—C48—O16	−18.8 (3)

Symmetry codes: (i) *x*−1, *y*, *z*; (ii) *x*+1, *y*, *z*; (iii) *x*, *y*−1, *z*; (iv) *x*, *y*+1, *z*.

### Hydrogen-bond geometry (Å, °)

**Table:**

*D*—H···*A*	*D*—H	H···*A*	*D*···*A*	*D*—H···*A*
O1W—H1A···O8^iv^	0.863 (18)	1.921 (19)	2.778 (3)	172 (4)
O1W—H1B···O4	0.863 (18)	1.874 (19)	2.733 (3)	174 (4)
N1—H1N···O2WA	0.901 (19)	2.03 (3)	2.808 (6)	144 (4)
N1—H1N···O2WB^v^	0.901 (19)	1.96 (3)	2.757 (5)	147 (4)
N2—H2N···O1W^i^	0.862 (17)	1.893 (18)	2.754 (3)	176 (3)
N3—H3N···O12^vi^	0.880 (17)	1.93 (2)	2.764 (3)	157 (3)

Symmetry codes: (iv) *x*, *y*+1, *z*; (v) −*x*, −*y*+2, −*z*; (i) *x*−1, *y*, *z*; (vi) −*x*−1, −*y*+1, −*z*+1.
